# Supplementary material for: Drug-Target Network Study Reveals the Core Target-Protein Interactions of Various COVID-19 Treatments
Source: Genes (Basel). 2022 Jul 6;13(7):1210. doi: 10.3390/genes13071210 (PMC9316565; doi:10.3390/genes13071210)
Supplement: Supplementary file 1 [file genes-13-01210-s001.zip › genes-1795211-Supplementary-Figures.pdf]

Article

Drug-Target Network Study Reveals the Core Target-Protein Interactions of Various COVID-19 Treatments

Yulin Dai [1,†], Hui Yu [2,†], Qiheng Yan [1,3,] Bingrui Li [1,4], Andi Liu [1,5], Wendao Liu [1,6], Xiaoqian Jiang [7], Yejin Kim [7], Yan Guo [2,*] and Zhongming Zhao [1,5,6,8,*]

Supplementary Materials

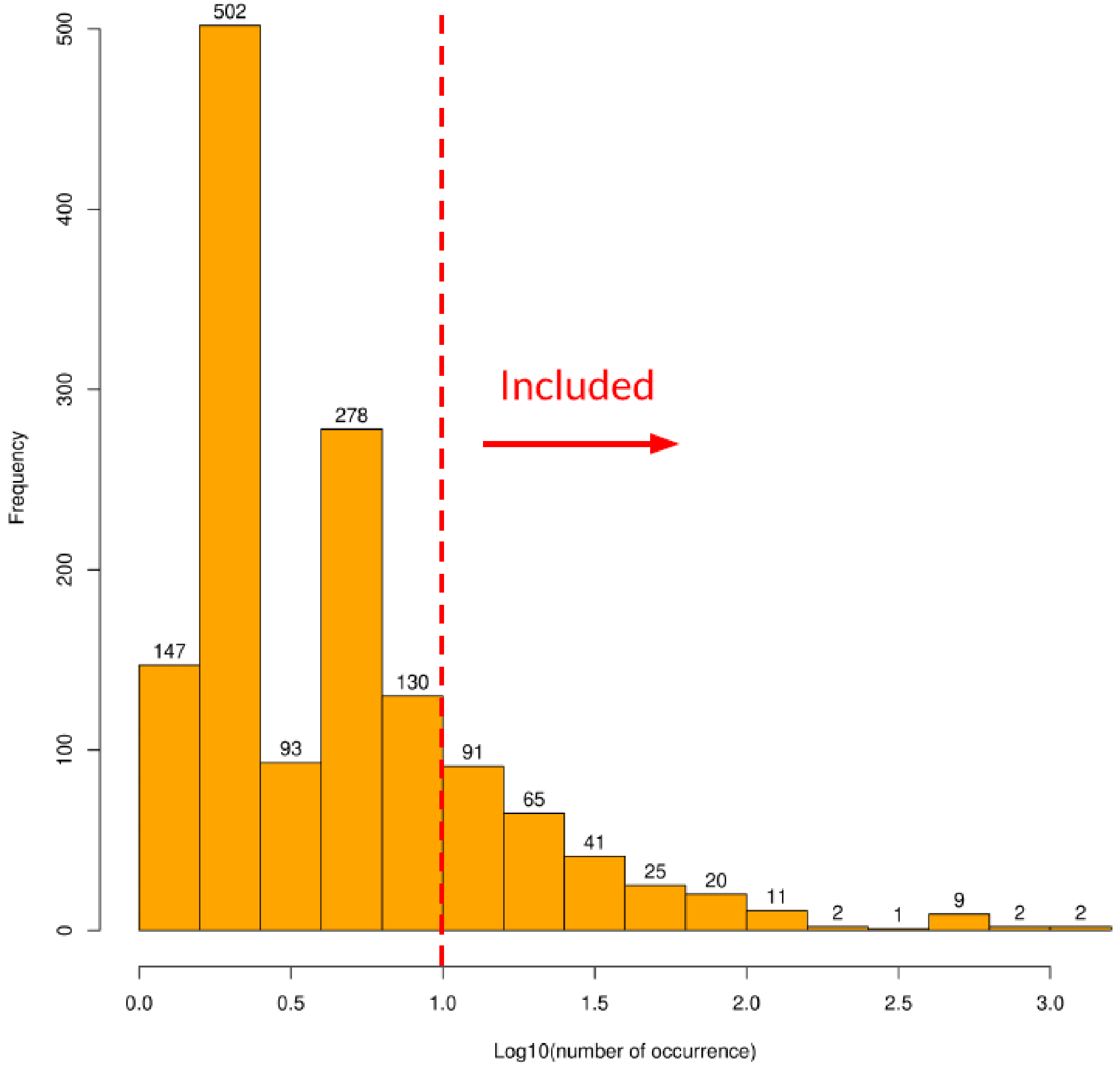

Figure S1. Overall distribution of the log10(frequency) for 1419 drugs. We included 1419 drugs with log10(frequency) > 1 from PubMed abstract mining.

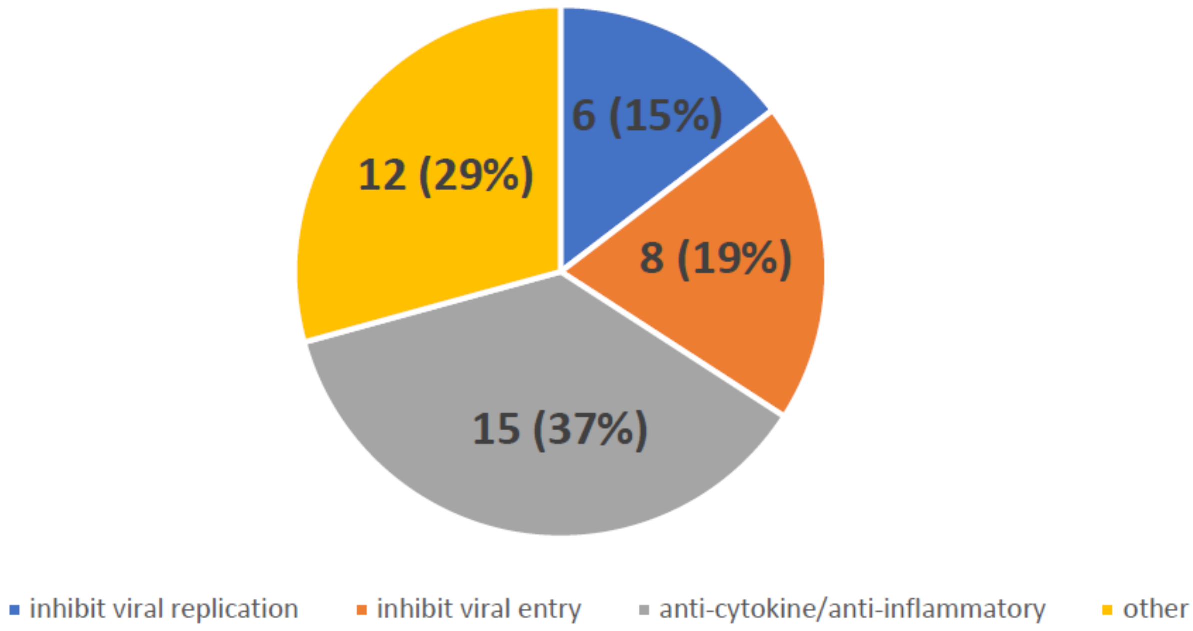

Figure S2. Pie chart shows the distribution of 41 COVID-19 drug with PharmGKB annotation.

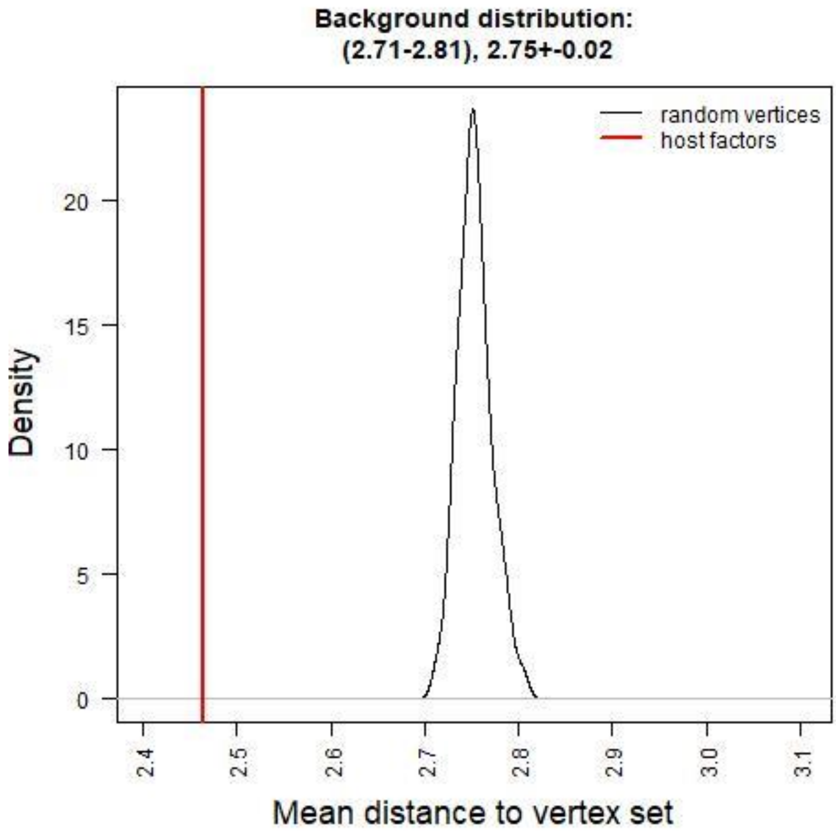

Figure S3. Distribution of 100 random sampling experiments of the mean shortest distance between any two genes in BioGRID. We plot the distribution of 100 random sampling experiments of the mean shortest distance between any two genes from random genes in BioGRID. The mean is 2.75 (se = 0.02). The range of mean is 2.71 (min) and 2.81 (max). The vertical red line is 2.46, which was calculated from the mean shortest distance between any two genes from drug targets and host factors in BioGRID.

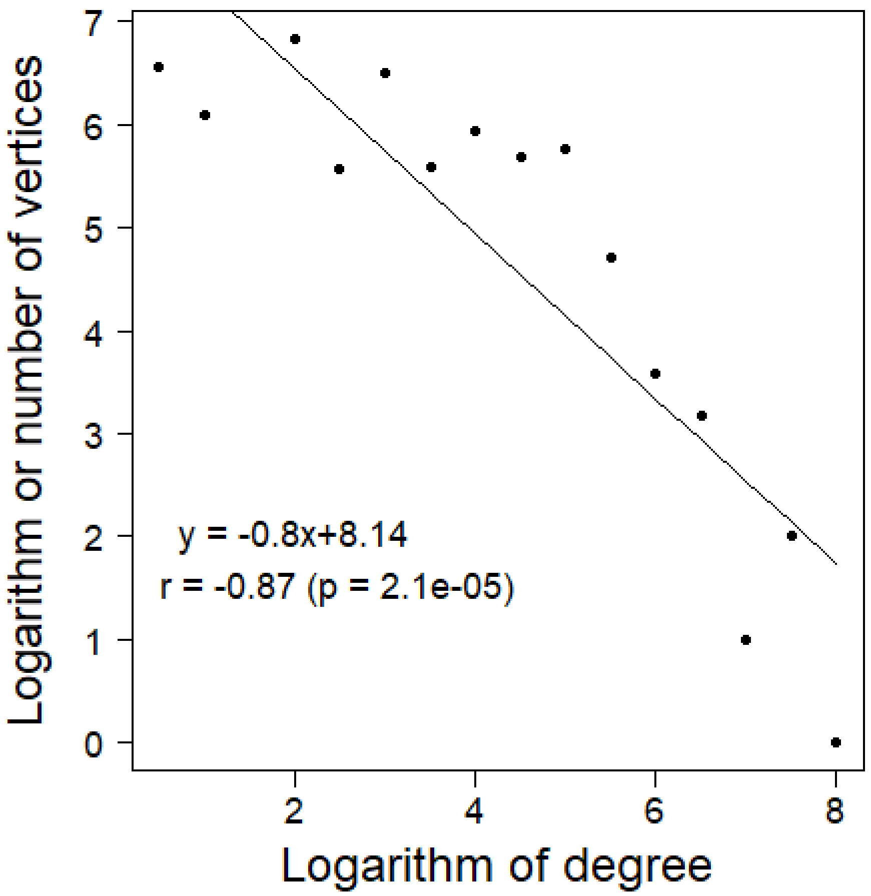

Figure S4. Scale-free property of our medium-scaled subnetwork consisting of 4,245 edges of 680 genes. Due to the sparsity of vertices degrees, we binned 680 genes into 15 categories by their log2 (vertices degree values) evenly (X-axis). Then, we calculated the total number of vertices in each binned category (Y-axis in log2 value). At the logarithm scale, the degree value and the frequency of the degree follows a linear relationship, indicating the scale-free property of a biological network.

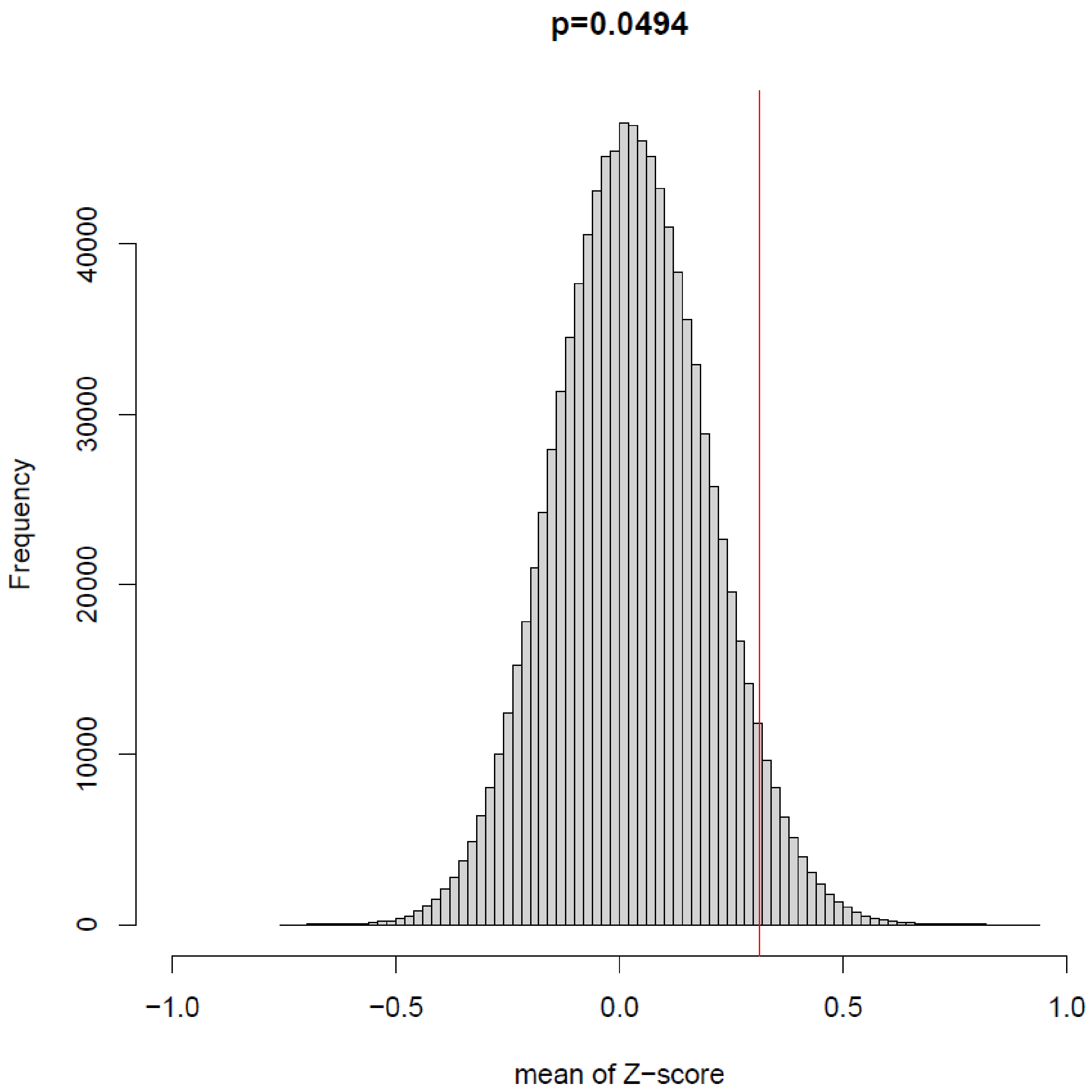

Figure S5. z-score permutation result for GWAS trait "Severe COVID-19 infection with respiratory failure (analysis I)". The red vertical line indicates the z-score permutation p value derived from 1 million times of permutations.
